# Brønsted acidity in zeolites measured by deprotonation energy

**DOI:** 10.1038/s41598-022-11354-x

**Published:** 2022-05-04

**Authors:** Michal Trachta, Roman Bulánek, Ota Bludský, Miroslav Rubeš

**Affiliations:** 1 Institute of Organic Chemistry and Biochemistry, Academy of Sciences of the Czech Republic, Flemingovo nám. 2, 162 10 Prague, Czech Republic; 2 Department of Physical Chemistry, Faculty of Chemical Technology, University of Pardubice, Studentská 573, 532 10 Pardubice, Czech Republic

**Keywords:** Theory and computation, Atomistic models, Electronic structure, Materials science, Porous materials

## Abstract

Acid forms of zeolites have been used in industry for several decades but scaling the strength of their acid centers is still an unresolved and intensely debated issue. In this paper, the Brønsted acidity strength in aluminosilicates measured by their deprotonation energy (DPE) was investigated for **FAU**, **CHA**, **IFR**, **MOR**, **FER**, **MFI**, and **TON** zeolites by means of periodic and cluster calculations at the density functional theory (DFT) level. The main drawback of the periodic DFT is that it does not provide reliable absolute values due to spurious errors associated with the background charge introduced in anion energy calculations. To alleviate this problem, we employed a novel approach to cluster generation to obtain accurate values of DPE. The cluster models up to 150 T atoms for the most stable Brønsted acid sites were constructed on spheres of increasing diameter as an extension of Harrison’s approach to calculating Madelung constants. The averaging of DPE for clusters generated this way provides a robust estimate of DPE for investigated zeolites despite slow convergence with the cluster size. The accuracy of the cluster approach was further improved by a scaled electrostatic embedding scheme proposed in this work. The electrostatic embedding model yields the most reliable values with the average deprotonation energy of about 1245 ± 9 kJ·mol^−1^ for investigated acidic zeolites. The cluster calculations strongly indicate a correlation between the deprotonation energy and the zeolite framework density. The DPE results obtained with our electrostatic embedding model are highly consistent with the previously reported QM/MM and periodic calculations.

## Introduction

Zeolites are crystalline microporous aluminosilicates that have a network of molecularly sized channels and cavities in their structure. Substitution of silicon with a trivalent element (most often Al, but it is also possible to incorporate Ga, Fe, B and other elements) creates a negative lattice charge compensated by extra-framework cations, which represent single, isolated active centers that give zeolites their unique catalytic, adsorption and ion-exchangeable properties. When the compensating cation is a proton, the zeolites become strong solid acids. The presence of these bridged hydroxyl groups, together with a large specific surface area, molecular sieve effect and high thermal stability, predestined zeolites to become one of the most important groups of heterogeneous catalysts and a cornerstone of the chemical industry. Zeolites, as strong solid acid catalysts, completely changed the face of the petrochemical industry in the second half of the twentieth century and became indispensable catalysts in oil, petrochemical, and fine chemical refining processes^1–3^.

**Figure 1:**
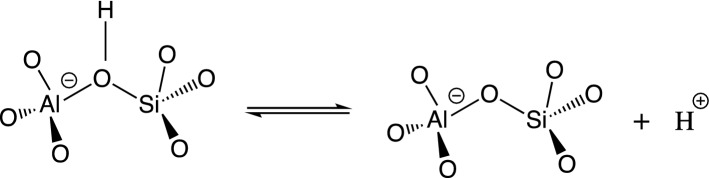
The Brønsted acidity of zeolites stems from the bridging hydroxyl groups.

Since most important industrial catalytic applications use the Brønsted acidity (i.e. H-forms) of zeolites, large effort has been devoted to the characterization of acid centers in zeolites, both in terms of number, accessibility and strength. The quantitative analysis is based on interaction of base molecules, usually nitriles, amines, pyridines, or phosphine oxides, with the acid sites monitored by IR, NMR, mass spectrometry, gas chromatography or temperature programmed techniques. The uptake of base molecules of various sizes probes the accessibility of acid sites. However, assessment of acid sites strength is the most complicated and discussed part of the characterization of acidic zeolite catalysts. The accurate assessment of zeolites’ Brønsted acidity is a long-standing issue due to the technical complexity on both theoretical and experimental parts, respectively^4^. The intrinsic acidity of the Brønsted acid sites (BAS) in zeolites can be determined via deprotonation energy (Fig. 1). The deprotonation energy (DPE) can be calculated directly by means of quantum chemistry methods. There are three main methodologies used (i) cluster approach^5–8^, (ii) hybrid quantum chemistry and molecular mechanics approach (QM/MM)^9–14^, and (iii) periodic DFT^15–17^. The calculated DPEs from the cluster approach suffer from the slow convergence with the cluster size, dependence on its termination, shape and geometry. There was an observation that electrostatic potential tends to converge for cluster sizes above ~ 20 tetrahedral units (20 T, where T = Si, Al)^5^. The QM/MM approach as proposed by Eichler et al.^9^ has shown an absolute accuracy of the DPE determination of about 10 kJ/mol with significantly decreased dependence on the cluster size within QM part. The more recent QM/MM studies by Rybicki and Sauer^13,14^ introduced different types of long-range corrections to DPE along with separating DPE into two components using classical Born model for proton solvation. One of the main conclusions was that DPE correlates with the inverse of the dielectric constant for 3-D and 2-D zeolites. To improve QM/MM accuracy a high degree of consistency between DFT and empirical potential calculations is required for the border region, which is by no means ensured. Especially, considering the slow convergence of the DPE with the cluster size. The periodic DFT provides reasonably accurate zeolite geometries and relative energies between different Brønsted acidity sites (BAS) particularly for large cell, where each of the BAS can be considered as an isolated site. Yet the energy of zeolitic anion is ill-defined because of the introduction of compensating background charge to converge electrostatic contribution of the “infinite” system. The current computational protocols to improve the periodic DFT energy of the charged defect seems to worsen the agreement with the cluster and QM/MM based results^15^.

In general, the DPE is experimentally not accessible in extended solids including zeolites. However, it can be investigated indirectly with various spectroscopy techniques (e.g. FT-IR, NMR), adsorption of probe molecules, and “test” reaction properties^18–27^. As a result, the comparison of zeolites Brønsted acidity between theory and experiment is highly problematic and still matter of debate. Nevertheless, there are some conclusions that can be drawn. Adsorption enthalpy of bases of increasing proton affinity (PA) lead to correlation with Brønsted acidity only for the zeolites with the same topology, thus distinguishing acidity strength between different framework topologies is difficult^28^. Also, size of the probe base molecule plays an important role due to the confinement inside the pores of different topologies (i.e. stability between the conjugate acid/adsorbate and conjugate base/framework), dispersion interactions, and possible diffusion limitations to BAS in zeolite channel or pockets accessible only through 8-memered rings^4,28–30^. The perturbation of BAS frequency (Δν_OH_) upon adsorption of weak bases (e.g. CO) was used to estimate the Brønsted acidity strength of various zeolites^24,25^. The correlation between heats of adsorption and change in Δν_OH_ frequency was established for several frameworks, but were shown not to be valid in general^24^. Moreover, the adsorption of weak bases samples only more stable regions of the BAS potential energy, thus insufficiently describes the deprotonation process itself. The proton transfers from BAS to reactant during reaction fulfills the very definition of Brønsted acidity. However, in case of reaction processes the DPE itself is not sufficient descriptor^31^. The reason is quite clear, for the reaction to procced the adsorption on BAS needs to take place and transition stated needs to be stabilized. Thus, the complexity of the problem rather increases than the opposite. On the other hand, there are several simple reactions such as H/D exchange, adsorbate protonation or water adsorption, which in principle should reflect the intrinsic acidity of BAS unless zeolite topology (i.e. confinement) starts to play an important role^20,21,27,29^.

In this work, the DPE is examined in detail for 7 different zeolite frameworks. These materials represent a reasonable sample from the zeolite database considering its: (i) dimensionality 1D (**IFR**, **TON**), 2D (**MOR**, **FER**), and 3D (**FAU**, **MFI**, **CHA**), (ii) size and shape of the entrance windows, and (iii) types of channels. Moreover, most of these zeolites are industrially important. The focus is on the accuracy of the determined DPEs, because the energy differences of about 6 kJ/mol can represent a change of one unit on the pKa scale^32^. Thus, even relatively “small” differences in DPEs between different BAS can have potentially important impact on theirs behavior. We propose a novel approach to a cluster generation to obtain absolute DPE values. The approach is based on DPE convergence of an average in series of clusters generated on spheres of increasing diameter. This approach can be considered as an extension of Harrison’s approach to calculate Madelung constants^33^.

## Methods

### Computational details

The periodic DFT calculations were performed with PBE functional with the plane wave (PW) energy cutoff of 400 eV^34^. The sampling of the first Brillouin zone was restricted to Γ-point due to the sufficiently large volumes of the investigated zeolites. The SCF energies and gradients were converged to 10^–7^ eV and 10^–3^ eV/Å, respectively. All periodic DFT calculations were performed with PAW pseudopotentials with ENMAX (O/400 eV, Si/245 eV, Al/240 eV, and H/250 eV) using the VASP package^35,36^. The cluster calculations were performed with the PBE functional using aug-cc-pVTZ (AVTZ) and def2-SVP basis sets using Turbomole code^37–39^.

### Structures

**Figure 2:**
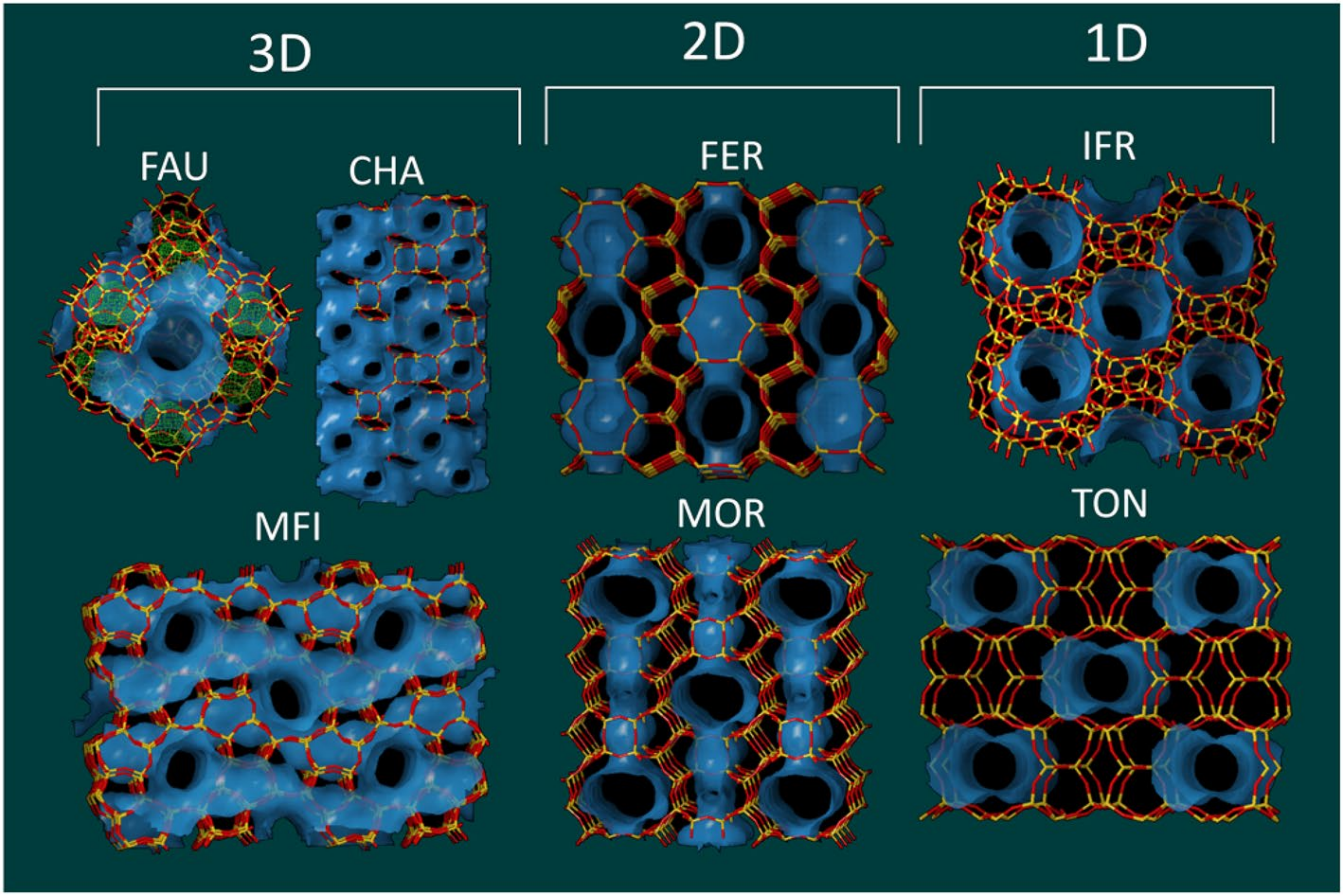
Structure and topology of investigated materials^44^.

The following structures were investigated: **FAU**, **CHA**, **IFR**, **MOR**, **FER**, **MFI,** and **TON** (Fig. 2). The unit-cell volumes were optimized in our previous work since the unit-cell size is a key descriptor for structural properties of purely siliceous zeolites^40^. In order to minimize BAS interactions in neighboring cells, *1* × *1* × *k* supercells were used for **IFR**, **MOR, FER (k** = 2)**,** and **TON (k** = 3). The corresponding BAS were created by replacing each unique Si position by Al, and thus creating negatively charged centers, which were compensated by proton. The protons were placed on each symmetrically inequivalent oxygen forming 110 structures in total. Each structure was run through simulated annealing from 600 K using modified SLC polarizable force-field in Gulp^41–43^. These structures present a good starting point for an ab initio optimization (see Ref.^40^). The relative stabilities of investigated structures are summarized in Table S1. The deprotonation energy of periodic model (*DPE*_periodic_) is defined as follows:

1 $${DPE}_{\text{periodic}}={E}_{\text{opt}}\left({\text{zeolite}}^{-}\right)-{E}_{\text{opt}}\left(\text{H-zeolite}\right)+\Delta ZPVE+\Delta BC,$$

where *E*_opt_(zeolite^−^) is energy of the optimized anionic structure after proton removal, *E*_opt_(H-zeolite) is energy of the optimized protonic form of zeolite, Δ*ZPVE* is the zero-point vibrational energy correction (zeolite^−^/H-zeolite), and Δ*BC* is a correction to compensating background charge described in Ref.^15^.

The clusters were generated for the most stable BAS for each investigated material (Table S1). The size of the clusters was limited up to about 150 T model (see Table S2). The cluster termination is done with silanol groups (r_OH_ = 0.97 Å) to keep the cluster composition as close as possible to original zeolite. The clusters are constructed in such a way that the distance of terminating hydrogens from the BAS proton is larger than a certain diameter generating spherically shaped nT models of increasing size. The cluster geometry is fixed at a periodic DFT geometry of the H-zeolite framework, thus only vertical deprotonation energies are calculated from the cluster models. Also, for larger clusters about 90 T the AVTZ calculations were no longer feasible and basis set correction (AVTZ/def2-SVP) has been employed (see Table S2). The vertical deprotonation energy of the *i*-th cluster model, ${DPE}_{i}^{vertical}$, is defined as follows:

2 $${DPE}_{i}^{\text{vertical}}={E}_{i}\left(\text{nT-anion}\right)-{E}_{i}\left(\text{nT-BAS}\right),$$

where *E*_*i*_(nT-BAS) is energy of the *i*-th BAS cluster model in periodic DFT geometry terminated with silanol groups and *E*_*i*_(nT-anion) is the energy of the corresponding anion in the same geometry. The cluster estimate of the BAS deprotonation energy, *DPE*_clusters_, is evaluated as an average DPE over all investigated nT clusters corrected for ZPVE and deformation energy:

3 $${DPE}_{{\text{c}}{\text{lusters}}}=\frac{{\sum }_{i=1}^{N}{DPE}_{i}^{\text{vertical}}}{N}-{E}_{\text{def}}+\Delta ZPVE,$$

where ${DPE}_{i}^{\text{vertical}}$ is defined in Eq. (2) , *N* is total number of investigated clusters for each BAS (Table S2), *E*_def_ is a deformation energy of an anion calculated from the periodic model and Δ*ZPVE* is the zero-point vibrational energy correction also calculated from the periodic model.

## Results and discussion

**Figure 3:**
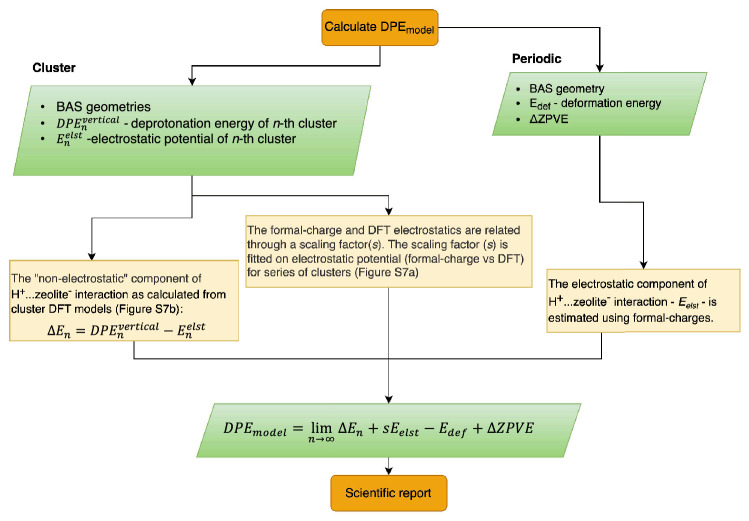
The electrostatic embedding model to calculate deprotonation energy (see also Figure S6).

The deprotonation energies for most stable BAS of investigated zeolites are summarized in Table 1. We present three DFT based methods for DPE evaluation: (i) the fully periodic calculations, *DPE*_periodic_, (ii) Harrison’s approach employing spherically shaped nT clusters with increasing diameter, *DPE*_clusters_, and (iii) scaled electrostatic embedding, *DPE*_model_, described in Fig. 3. The most direct but somewhat problematic approach to determining DPE is a fully periodic DFT calculation. Introducing the compensating background charge into the periodic calculation to avoid the divergence of the Coulomb term causes a sizable error in zeolitic anion energy. Thus, the *DPE*_periodic_ needs to be vertically shifted because absolute values are significantly off. It has also been shown that the periodic DPE error correlates well with the framework density (FD) (Figure S2)^15^. Using the linear correlation as shown in Figure S2 the corrected *DPE*_periodic_ should be close to the DPE estimates calculated by other methods. The standard deviation of deprotonation energy between investigated zeolites corresponds to 6 kJ·mol^-1^ with maximum difference of 18 kJ·mol^-1^ between **CHA** and **FER** materials. It seems that differences between various zeolites are quite on par with differences between sites within a zeolite framework. For example, the **MFI** framework has twelve distinct T-positions and the standard deviation of the deprotonation energy, *DPE*_periodic_, from its mean is about 5 kJ·mol^-1^. The agreement between our periodic results and those of Ref.^15^ is quite reasonable (cf. Figure S3) considering that different functional and unit-cell geometries were used. The most notable difference is that our Δ*BC* correction slightly differs from the one used in Ref.^15^ as we do not observe any outlier values of calculated DPE, *e.g.* for the Al4 site in the **MOR** zeolite. The most probable cause of this discrepancy is that some of the geometries used in previous work correspond to local minima on the potential energy surface that are too high in energy.

**Table 1:** The deprotonation energy (in kJ·mol^-1^) for most stable BAS along with correction to zero-point vibrational energy and anion deformation energy (in kJ·mol^-1^) from periodic model calculations.

Zeolite	BAS	ΔZPVE	E_def_	DPE_periodic_^a^	DPE_clusters_^b^	DPE_model_^c^
FAU	Al1-O1	−29^d^	117	1248	1208 (11)	1210
CHA	Al1-O4	−29	107	1231	1234 (11)	1237
IFR	Al1-O5	−29	102	1250	1243 (10)	1250
	Al2-O1	−29	109	1236	1230 (11)	1236
	Al3-O5	−29	102	1244	1236 (8)	1244
	Al4-O9	−29	106	1244	1234 (9)	1249
MOR	Al1-O4	−30	117	1241	1237 (7)	1233
	Al2-O7	−30	117	1243	1237 (5)	1235
	Al3-O3	−28	113	1242	1239 (14)	1242
	Al4-O7	−28	115	1248	1245 (7)	1240
FER	Al1-O3	−29	113	1243	1245 (9)	1246
	Al2-O2	−28	110	1251	1254 (14)	1256
	Al3-O7	−28	113	1252	1252 (5)	1254
	Al4-O7	−29	119	1249	1248 (5)	1250
MFI	Al1-O2	−28	114	1250	1240 (12)	1245
	Al2-O2	−29	113	1253	1241 (7)	1251
	Al3-O9	−28	127	1249	1241 (6)	1246
	Al4-O4	−29	112	1253	1242 (8)	1254
	Al5-O12	−28	116	1247	1235 (9)	1246
	Al6-O13	−29	117	1245	1234 (12)	1243
	Al7-O17	−29	111	1245	1231 (10)	1244
	Al8-O17	−29	112	1249	1239 (6)	1251
	Al9-O21	−28	120	1242	1232 (13)	1240
	Al10-O24	−26	126	1239	1231 (13)	1241
	Al11-O24	−29	107	1250	1242 (13)	1253
	Al12-O26	−29	119	1237	1228 (14)	1237
TON	Al1-O2	−30	117	1244	1252 (5)	1258
	Al2-O3	−29	112	1234	1241 (8)	1248
	Al3-O4	−29	110	1239	1246 (9)	1248
	Al4-O2	−29	111	1240	1247 (4)	1254

^a^See Eq. (1), vertical shift is set to 1243 kJ·mol^−1^ to yield same mean value as DPE_model_.

^b^See Eqs. (2–3), averaging is performed for cluster 9 T onward (see Figure S4) and standard deviation is given in paratheses.

^c^See Fig. 3 for definition of *DPE*_model_.

^d^Due to the **FAU** unit-cell size the mean value of ΔZVPE from other calculations was taken as an estimate.

The cluster approach enables accessing the deprotonation energy in a straightforward fashion, *i.e.* there are no uncertainties in energies (vertical shifts), but the convergence of DPE with the cluster size possess its own set of problems. The results for clusters up to the 150 T model are summarized in Table 1. The slow convergence of deprotonation energy with the cluster size is manifested by large standard deviations for most of the investigated BAS. The average value of deprotonation energy, *DPE*_clusters_, of all investigated zeolites is determined to be 1239 ± 10 kJ·mol^-1^. Although, it may seem that difference between various materials is quite small, the standard deviation spams about two units on pKa scale and maximum difference between cluster deprotonation energies is 46 kJ·mol^-1^ (**FAU**/**FER**(T2)—Table 1).

**Figure 4:**
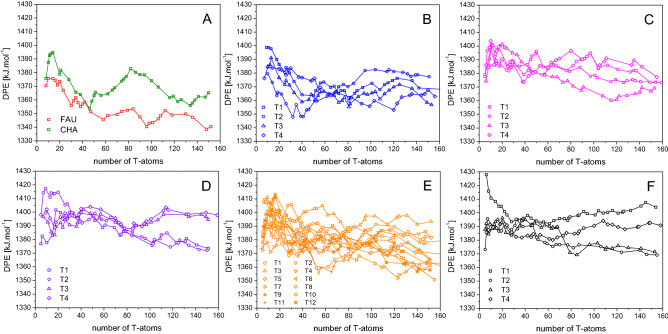
The vertical deprotonation energy dependence on cluster size for each investigated zeolite; (**A**) **FAU** and **CHA** zeolite, (**B**) **IFR**, (**C**) **MOR**, (**D**) **FER**, (**E**) **MFI**, (**F**) **TON**.

**Figure 5:**
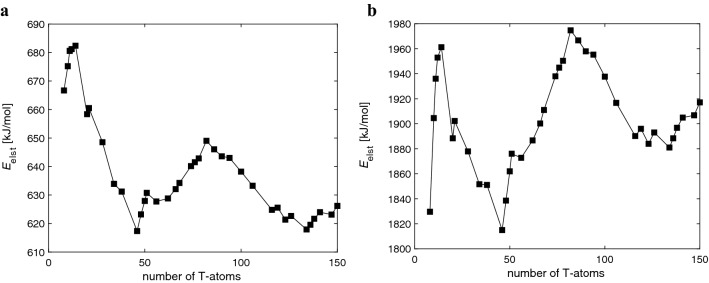
Comparison between (**a**) PBE/def2-SVP electrostatic potential and (**b**) electrostatic potential calculated from formal charges for model clusters of **CHA**.

The Fig. 4 shows vertical deprotonation energies, ${DPE}_{i}^{\text{vertical}}$, as a function of cluster size for all investigated materials and Al-positions and variance for each nT cluster is plotted in Figure S4. The small drift in the data can be noticed; it indicates that even clusters with size about ~ 150 T do not provide fully converged results. This observation is not that surprising considering the long-range nature of electrostatic interactions. The *DPE*_clusters_ values show quite similar energy differences between different Al-positions in a zeolites framework (*e.g.* **MFI**) as results obtained from periodic calculations; the average absolute deviation is only 1 kJ·mol^-1^. This observation indicates a highly consistent energetics obtained from our cluster and periodic calculations. Note that the vertical shift is the same for all Brønsted sites within a given zeolite framework. The unfavorable dependence of DPE on cluster size can be alleviated only through an empirical model, *e.g.* various QM/MM approaches^9–14^. In this paper we employ the scaled electrostatic embedding (Fig. 3). The deprotonation energies obtained by this approach, *DPE*_model_, are reported in Table 1. Figure 5 shows comparison between electrostatic potential (*E*_elst_) at the BAS hydrogen position calculated at ab initio level and from formal charges for **CHA**^**−**^ clusters (cf. Figure S1). Quite similar graphs can be obtained for other zeolitic structures, although their behavior can be substantially more oscillating. The electrostatic potential calculated from formal charges can capture the shape, but its absolute value and amplitudes of changes are significantly overestimated for obvious reasons (electronic overlap, polarization, screening effects, etc*.*). However, there is a strong indication of scalability between the empirical (formal charges) and ab initio *E*_elst_ potentials. Based on these observations the Fig. 3 depicts a general idea behind the correction scheme, on which the calculated deprotonation energy, *DPE*_model_, is based. The detailed description of the method is provided in Supporting information.

**Figure 6:**
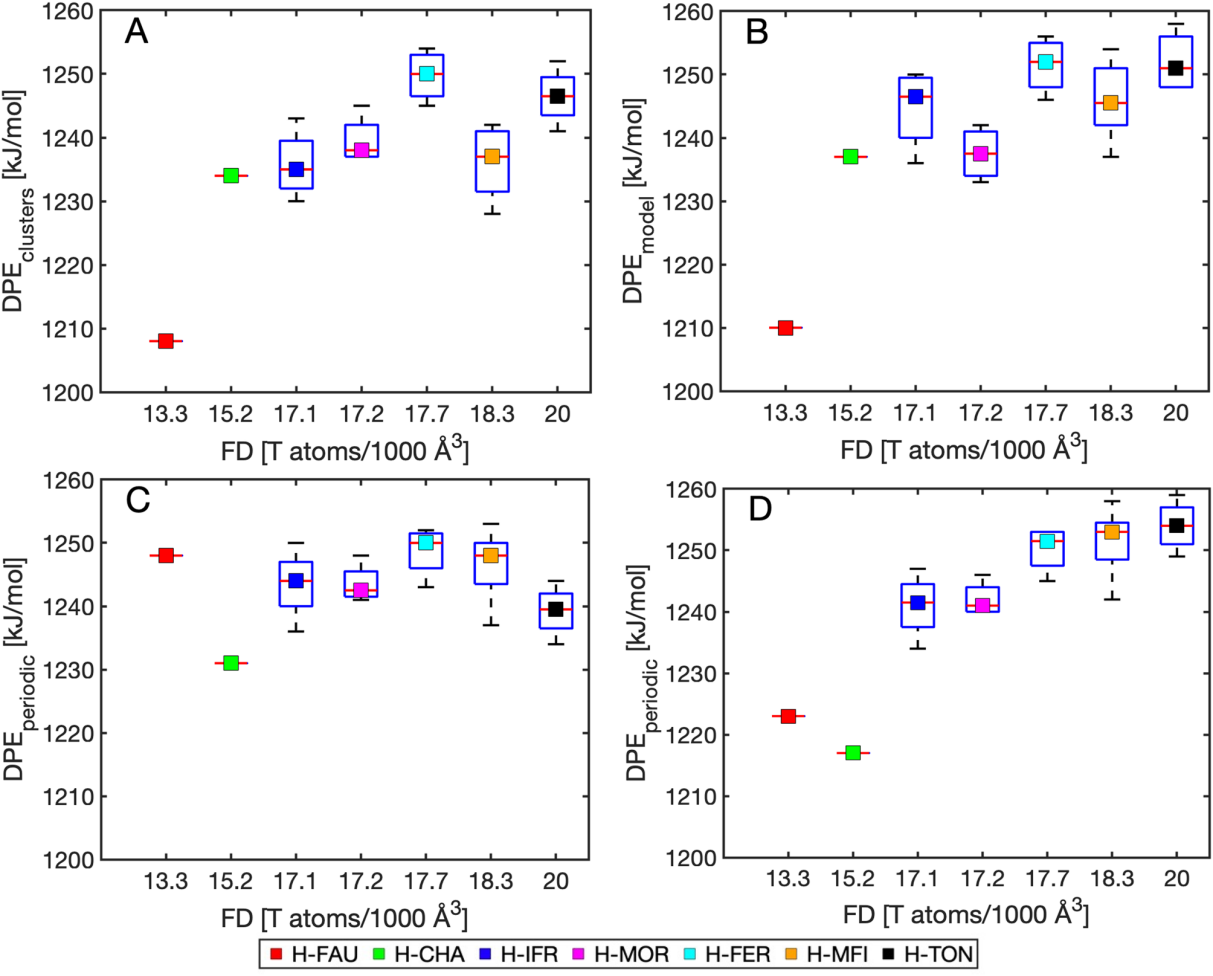
Deprotonation energies of investigated materials (**A**) *DPE*_clusters_, (**B**) *DPE*_model_, (**C**) *DPE*_periodic_ with ∆*BC* defined in Eq. 1 with vertical shift to yield the same mean as *DPE*_model_, and (**D**) *DPE*_periodic_ with ∆BC taken as an error from *DPE*_model_ as shown in Figure S5. The boxplots show statistical behavior within material themselves.

The average value of deprotonation energy of investigated zeolites calculated from the *DPE*_model_ values in Table 1 yields 1245 ± 9 kJ·mol^-1^, which does not significantly differ from the values determined from cluster calculations (1239 ± 10 kJ·mol^-1^). However, the subtle changes between the models can be observed as demonstrated in Fig. 6. The deprotonation energies calculated from the electrostatic embedding (*DPE*_model_) show a correlation between framework density and deprotonation energy. This would suggest that increased framework density tend to improve stabilization of zeolitic anion and on average slightly decrease the Brønsted acidity of the material. This conclusion is further supported by recent QM/MM calculations for various 3-D zeolites, where the correlation of DPE with the inverse of dielectric constant (i.e. T-site density) was observed^13^. The differences between *DPE*_model_ and *DPE*_cluster_ reflect the slow convergence of the deprotonation energy with cluster size mainly for the **IFR** and **MFI** frameworks (Fig. 6). As mentioned before the *DPE*_periodic_ values are subject to large uncertainties associated with the Δ*BC* correction (Eq. 1) and should be compared against *DPE*_model_ and *DPE*_cluster_ values with caution. In this work we used the vertical shift of the periodic DPE values in such a way that mean deprotonation energy over all the frameworks and sites in Table 1 is the same as for *DPE*_model_ (i.e. 1245 kJ·mol^−1^) (Fig. 6C, D). This choice of the vertical shift allows direct comparison with cluster-based approaches. In Fig. 6D the dependence of the Δ*BC* correction on the framework density was adjusted to our *DPE*_model_ values. As can be seen from Figs. 6A, B the results of cluster-based methods, *i.e.* without employing the background charge in the DPE evaluation, also depend on the framework density to some extent. Thus, a complete removal of the *DPE*_periodic_ dependence on framework density as suggested in Ref.^15^ (Fig. 6C) seems to lead to a background charge overcompensation in periodic calculations. The most marked differences between DPEs calculated from periodic models and electrostatic embedding results are observed for the **CHA** and **FAU** frameworks. The possible explanation is limited validity of the linear *∆BC* correction for materials with very low framework density. Note that QM/MM calculations also predicted the lowest DPE for the **CHA** (1190 kJ·mol^−1^) and **FAU** (1171 kJ·mol^−1^) frameworks^10^.

The comparison with QM/MM literature data is summarized in Tables S3 and S4. The agreement can be considered as quite reasonable upon considering the importance of following issues: (i) convergence of the DPE results with the basis set size, (ii) accuracy of the ΔZVPE correction, (iii) method employed for the DPE evaluation such as Hartree–Fock or DFT with hybrid functionals. The AVTZ basis set used in this work is sufficiently large to provide accurate description mainly for the anion, where diffuse functions are essential, and thus the cluster calculation in this work can be regarded as reasonably converged with respect to the basis set size. The ΔZPVE correction is obtained from the periodic model within harmonic approximation, and thus the correction is slightly overestimated. On the other hand, the variance in ΔZPVE is quite small and thus the ΔZPVE can be considered as nearly constant (about −29 kJ·mol^−1^). Using different computational methods and schemes (*e.g.* basis set incompleteness correction) would likely yield a small vertical shift in the calculated DPEs, but we expect that the observed trends remain valid. This conclusion is further supported by comparing calculated DPEs with values in Ref.^13^ as shown in Table S4. The DPEs are on average shifted by 26 kJ·mol^−1^ with a standard deviation of 9 kJ·mol^−1^. Thus, reasonable consistency between the data calculated by two markedly different methodologies is observed. The uncertainty in *DPE*_model_ values is difficult to predict accurately, however, it can be expected that standard deviation should be significantly decreased compared to *DPE*_cluster_ standard deviations (Table 1).

## Conclusions

The deprotonation energy as a convenient descriptor of the intrinsic Brønsted acid strength of the aluminosilicate zeolites was investigated by means of periodic and cluster DFT calculations. The intrinsic acidity measured by the deprotonation energy is a theoretical concept that bypasses the complicated interpretation of probe molecule adsorption. On the other hand, the accurate assessment of DPE in extended systems is challenging for contemporary computational chemistry, especially considering the accuracy required to obtain deprotonation energies within one unit of the pKa scale. The scaled electrostatic embedding model proposed in this work has shown that the Brønsted acidity strength of aluminosilicates inversely correlates with framework density (i.e., with increasing density, the Brønsted acidity strength decreases). This observation is supported by a series of cluster calculations of increasing size up to 150 T. The clusters were constructed as an extension of Harrison’s approach to calculating Madelung constants. Due to the highly oscillating nature of DPE, the focus is on the convergence of the mean on a series of cluster models rather than taking a single value of deprotonation energy from a particular cluster calculation. This “brute force” approach provides surprisingly robust estimates of DPE consistent with the electrostatic embedding model and periodic results. Differences in Brønsted acidity strength for different Al-positions within materials themselves are far from being negligible. The **MFI** can be an illustrative example, where the difference between T4 and Tl2 Brønsted acid sites spans 14–17 kJ·mol^−1^. Consequently, the catalytic activity of the aluminosilicates, besides their topology, is likely to be also influenced by the Al-distribution of “real” samples.

## Supplementary Information

Supplementary Information 1.

Supplementary Information 2.

## Data Availability

All investigated structures are included as zip archive (structures.zip) in the Supporting information. All data used in the current study are available from the corresponding author on reasonable request.
