# Supplementary material for: Brønsted acidity in zeolites measured by deprotonation energy
Source: Sci Rep. 2022 May 4;12:7301. doi: 10.1038/s41598-022-11354-x (PMC9068704; doi:10.1038/s41598-022-11354-x)
Supplement: Supplementary file 1 — Supplementary Information 1. [file 41598_2022_11354_MOESM1_ESM.pdf]

Supporting information:
Brønsted acidity in zeolites measured by deprotonation energy

Michal Trachta[1], *Roman Bulánek*[2], *Ota Bludský*[1], *Miroslav Rubeš* [1*]

[1]Institute of Organic Chemistry and Biochemistry, Academy of Sciences of the Czech Republic, Flemingovo nám. 2, 162 10 Prague, Czech Republic

[2] Department of Physical Chemistry, Faculty of Chemical Technology, University of Pardubice, Studentská 573, 532 10 Pardubice, Czech Republic

E-mail: miroslav.rubes@uochb.cas.cz

Table S1 The stability of different BAS of investigated zeolites along with some basic geometry parameters (r_{O-H} – BAS bond length, r_{Al-H} – BAS distance from Al, $r_{O(Si)-H}$ – the closest BAS distance from oxygen connected to Si as indicator of intra-zeolitic hydrogen bond, and T-O-T angle defined as angle between Al-O-Si of the BAS see also Figure 1). The BAS numbering corresponds to IZA – the **H-TON** a,b indexes at O6 reflect the lower symmetry (#36) of the experimental structure.

BAS	Total energy[eV]	r_{O-H}[Å]	r_{Al-H}[Å]	$r_{O(Si)-H}$[Å]	T-O-T [deg]	E_{rel}[kJ/mol]
**H-FAU (a = b = c = 24.3351 Å α = β = γ = 90°)**						
Al1-O1	-4558.63800	0.97541	2.45	2.63	127.1	0.0
Al1-O2	-4558.61290	0.9795	2.52	2.66	127.4	2.4
Al1-O3	-4558.55550	0.98103	2.46	2.60	131.0	7.9
Al1-O4	-4558.56490	0.97647	2.42	2.54	130.4	7.0
**H-CHA (a = b = 13.6593 Å c = 13.6500 Å α = β = 90° γ = 120°)**						
Al1-O1	-856.33606	0.97670	2.47	2.63	131.5	4.8
Al1-O2	-856.32298	0.97794	2.37	2.71	133.8	6.0
Al1-O3	-856.36294	0.97786	2.39	2.57	134.9	2.2
Al1-O4	-856.38568	0.97612	2.45	2.65	130.9	0.0
**H-IFR (a = 18.5277 Å b = 13.5815 Å c = 15.2237 Å α = 90° β = 101.8° γ = 90°)**						
Al1-O3	-1520.48330	1.00913	2.38	1.74	135.4	8.2
Al1-O5	-1520.56880	0.99466	2.55	1.94	123.6	0.0
Al1-O8	-1520.45800	0.97513	2.46	2.58	127.3	10.7
Al1-O9	-1520.47960	0.98342	2.50	2.35	131.0	8.6
Al2-O1	-1520.49750	0.99337	2.44	1.89	136.0	0.0
Al2-O2	-1520.32970	0.99459	2.47	1.93	127.3	16.2
Al2-O3	-1520.45030	1.00160	2.35	1.81	134.5	4.6
Al2-O4	-1520.44300	0.97739	2.40	2.85	134.2	5.3
Al3-O1	-1520.57620	0.99710	2.38	1.86	134.1	0.8
Al3-O5	-1520.58400	0.99021	2.45	2.06	125.4	0.0
Al3-O6	-1520.50280	0.97944	2.49	2.62	128.9	7.8
Al3-O7	-1520.30730	1.00278	2.36	1.82	132.2	26.7
Al4-O7	-1520.30310	0.99246	2.49	1.95	133.4	19.2
Al4-O8	-1520.46760	0.97550	2.43	2.73	126.3	3.3
Al4-O9	-1520.50160	0.98331	2.46	2.45	129.1	0.0
Al4-O10	-1520.37970	0.97754	2.39	2.48	134.2	11.8
**H-MOR (a = 18.1261 Å b = 20.4853 Å c = 15.0409 Å α = β = γ = 90°)**						
Al1-O1	-2281.81440	0.97772	2.41	2.68	135.8	5.9
Al1-O2	-2281.79940	0.97777	2.41	2.61	137.0	7.4
Al1-O3	-2281.84130	0.97622	2.50	2.60	128.4	3.3
Al1-O4	-2281.87580	0.97798	2.41	2.90	134.0	0.0
Al2-O4	-2281.78320	0.97731	2.46	2.49	137.0	7.3
Al2-O5	-2281.83820	0.97730	2.41	2.57	135.4	2.0
Al2-O6	-2281.74540	1.02044	2.35	1.62	147.6	10.9
Al2-O7	-2281.85850	0.97657	2.43	2.52	132.7	0.0
Al3-O3	-2281.86010	0.97592	2.48	2.79	128.0	0.0
Al3-O8	-2281.77770	0.97827	2.39	2.56	136.2	8.0
Al3-O9	-2281.72390	0.99819	2.45	1.79	141.6	13.1
Al4-O7	-2281.79770	0.97597	2.45	2.65	129.8	0.0

Al4-O9	-2281.65280	1.00575	2.36	1.71	138.5	14.0
Al4-O10	-2281.71110	0.97874	2.37	2.50	135.2	8.4
**H-FER (a = 19.0008 Å b = 14.2151 Å c = 15.0194 Å α = β = γ = 90°)**						
Al1-O1	-1711.93660	0.97716	2.46	2.43	136.3	4.4
Al1-O2	-1711.97170	0.97781	2.47	2.62	130.0	1.0
Al1-O3	-1711.98250	0.97775	2.40	2.57	136.6	0.0
Al1-O4	-1711.86410	1.00966	2.38	1.71	147.9	11.4
Al2-O2	-1712.02480	0.97766	2.47	2.72	131.2	-7.2
Al2-O5	-1711.94970	0.97894	2.42	2.48	137.7	0.0
Al2-O6	-1711.83310	0.98095	2.32	2.51	144.9	11.3
Al3-O1	-1712.01570	0.97663	2.43	2.91	131.2	2.4
Al3-O7	-1712.04060	1.00128	2.49	1.76	133.8	0.0
Al3-O8	-1711.91130	0.97805	2.39	2.55	138.1	12.5
Al4-O5	-1712.02390	0.98226	2.42	2.44	134.9	2.2
Al4-O7	-1712.04640	1.00172	2.39	1.79	133.0	0.0
**H-MFI (a = 19.8539 Å b = 19.8700 Å c = 13.2913 Å α = β = γ = 90°)**						
Al1-O1	-2281.03830	0.97888	2.36	2.44	139.6	7.9
Al1-O2	-2281.12010	0.97563	2.46	2.85	122.1	0.0
Al1-O3	-2281.07010	0.97619	2.46	2.55	132.7	4.8
Al1-O4	-2280.94780	1.00699	2.37	1.75	144.3	16.6
Al2-O2	-2281.39030	0.9743	2.48	2.73	125.0	-3.6
Al2-O5	-2281.22500	0.9765	2.42	2.56	132.0	12.3
Al2-O6	-2281.24110	0.97924	2.42	2.74	138.9	10.8
Al2-O7	-2281.35280	1.01284	2.38	1.63	131.4	0.0
Al3-O5	-2281.25350	0.97701	2.39	2.54	134.3	8.1
Al3-O8	-2281.31370	0.9764	2.42	2.78	131.6	2.3
Al3-O9	-2281.33730	1.02235	2.38	1.58	139.0	0.0
Al3-O10	-2281.29560	1.0103	2.36	1.72	142.0	4.0
Al4-O4	-2281.24130	1.01765	2.39	1.62	136.9	-12.0
Al4-O9	-2281.00310	0.99668	2.40	1.88	137.5	11.0
Al4-O11	-2281.11670	0.98086	2.39	2.65	134.6	0.0
Al4-O12	-2281.06910	0.97978	2.35	2.91	136.8	4.6
Al5-O1	-2281.02580	0.9754	2.45	2.73	126.7	13.2
Al5-O12	-2281.16290	0.99393	2.42	1.93	132.5	0.0
Al5-O13	-2281.08030	0.97777	2.39	2.47	137.2	8.0
Al5-O14	-2281.06630	1.01607	2.37	1.65	138.8	9.3
Al6-O7	-2281.26500	0.99165	2.44	1.98	129.6	2.2
Al6-O10	-2281.00380	1.00726	2.39	1.72	136.8	27.4
Al6-O13	-2281.28800	0.97651	2.40	2.90	132.8	0.0
Al6-O15	-2281.25590	0.97724	2.40	2.59	131.0	3.1
Al7-O11	-2281.09210	0.97772	2.42	2.94	134.8	11.3
Al7-O16	-2281.11900	0.97872	2.36	2.89	135.5	8.7

Al7-O17	-2281.20940	0.97504	2.52	2.57	125.7	0.0
Al7-O18	-2281.00970	0.97994	2.35	2.47	141.8	19.3
Al8-O6	-2281.29220	0.99935	2.43	1.83	136.9	8.9
Al8-O17	-2281.38460	1.00386	2.46	1.72	135.9	0.0
Al8-O19	-2281.13910	0.98206	2.30	2.89	139.9	23.7
Al8-O20	-2281.18730	1.01227	2.38	1.69	142.4	19.0
Al9-O15	-2281.15790	0.97616	2.41	2.97	123.9	2.3
Al9-O19	-2281.16420	0.98293	2.41	2.44	136.2	1.7
Al9-O21	-2281.18220	0.97675	2.40	2.53	133.2	0.0
Al9-O22	-2280.94740	0.99661	2.38	1.89	138.8	22.7
Al10-O3	-2281.10520	0.97654	2.42	2.82	131.2	6.0
Al10-O22	-2280.99810	1.001	2.46	1.76	137.8	16.3
Al10-O23	-2281.08800	0.97501	2.46	2.69	125.6	7.6
Al10-O24	-2281.16710	1.00904	2.40	1.72	136.1	0.0
Al11-O14	-2281.09730	1.00488	2.47	1.72	137.0	22.3
Al11-O16	-2280.97950	0.97941	2.34	2.83	137.5	33.7
Al11-O24	-2281.32870	1.00284	2.48	1.75	136.9	0.0
Al11-O25	-2281.28320	0.98702	2.44	2.12	137.0	4.4
Al12-O8	-2281.28160	0.97542	2.47	2.54	130.3	3.0
Al12-O20	-2281.28100	1.00351	2.38	1.77	144.9	3.0
Al12-O25	-2281.28590	0.97491	2.49	2.60	128.9	2.6
Al12-O26	-2281.31240	0.97972	2.35	2.51	139.5	0.0
**H-TON (a = 13.8689 Å b = 17.3715 Å c = 14.9431 Å α = β = γ = 90°)**						
Al1-O1	-1711.72620	0.97918	2.45	2.49	128.4	9.1
Al1-O2	-1711.82100	1.01086	2.42	1.72	140.7	0.0
Al1-O3	-1711.75600	0.97653	2.45	2.72	131.8	6.3
Al2-O3	-1711.92770	0.97745	2.40	2.83	130.0	-5.6
Al2-O4	-1711.77430	0.97886	2.36	2.57	137.6	9.2
Al2-O5	-1711.86930	1.00019	2.42	1.83	133.2	0.0
Al3-O4	-1711.91910	0.97694	2.40	2.66	134.0	-18.3
Al3-O6a	-1711.72940	0.98204	2.46	2.38	133.0	0.0
Al3-O6b	-1711.69720	0.98302	2.46	2.30	133.0	3.1
Al4-O2	-1711.86780	0.99876	2.46	1.81	132.2	-3.5
Al4-O6a	-1711.75890	0.98228	2.48	2.29	131.8	7.0
Al4-O6b	-1711.83180	0.98330	2.44	2.33	135.2	0.0

Table S2 The number (n) and sizes (nT – number of T atoms) of investigated clusters at PBE/def2-SVP level of theory. The basis set correction in kJ/mol is defined as a difference between vertical DPE at PBE/AVTZ and PBE/def2-SVP levels of theory for the largest common cluster (number in paratheses), respectively (see also Figure S1). The last column defines the minimum cluster size for which the electrostatic fit meets the rmsd criteria of 5.7 kJ/mol, which corresponds to one unit of pKa scale.

Zeolite	BAS	n	nT	basis set corr.	nT(electrostatic fit)
**FAU**	Al1-O1	33	8-152(90)	-38	58
**CHA**	Al1-O4	37	8-150(94)	-47	50
**IFR**	Al1-O5	33	9-164(93)	-48	81
	Al2-O1	32	10-151(95)	-51	76
	Al3-O5	35	10-153(94)	-49	77
	Al4-O9	31	7-156(92)	-45	65
**MOR**	Al1-O4	36	5-154(88)	-51	50
	Al2-O7	34	5-159(92)	-54	57
	Al3-O3	33	6-153(91)	-48	37
	Al4-O7	32	6-155(94)	-52	53
**FER**	Al1-O3	32	5-151(85)	-52	39
	Al2-O2	34	5-151(96)	-52	34
	Al3-O7	32	7-151(88)	-51	56
	Al4-O7	29	9-159(95)	-51	48
**MFI**	Al1-O2	28	5-151(66)	-53	45
	Al2-O2	30	5-151(90)	-50	45
	Al3-O9	30	7-152(80)	-52	50
	Al4-O4	33	7-152(85)	-51	68
	Al5-O12	31	6-162(86)	-50	73
	Al6-O13	33	5-163(74)	-53	53
	Al7-O17	31	5-155(89)	-48	41
	Al8-O17	33	9-152(94)	-47	94
	Al9-O21	30	6-170(86)	-52	42
	Al10-O24	30	7-171(95)	-49	82
	Al11-O24	30	9-151(90)	-48	80
	Al12-O26	30	5-151(80)	-50	67
**TON**	Al1-O2	40	6-154(79)	-53	78
	Al2-O3	30	5-155(70)	-54	46
	Al3-O4	36	5-153(68)	-55	48
	Al4-O2	36	8-158(86)	-54	76

Figure S1 The basis set dependence of **CHA** cluster models (a) indicates that with increasing size the difference between different basis sets converge to a nearly constant (b).

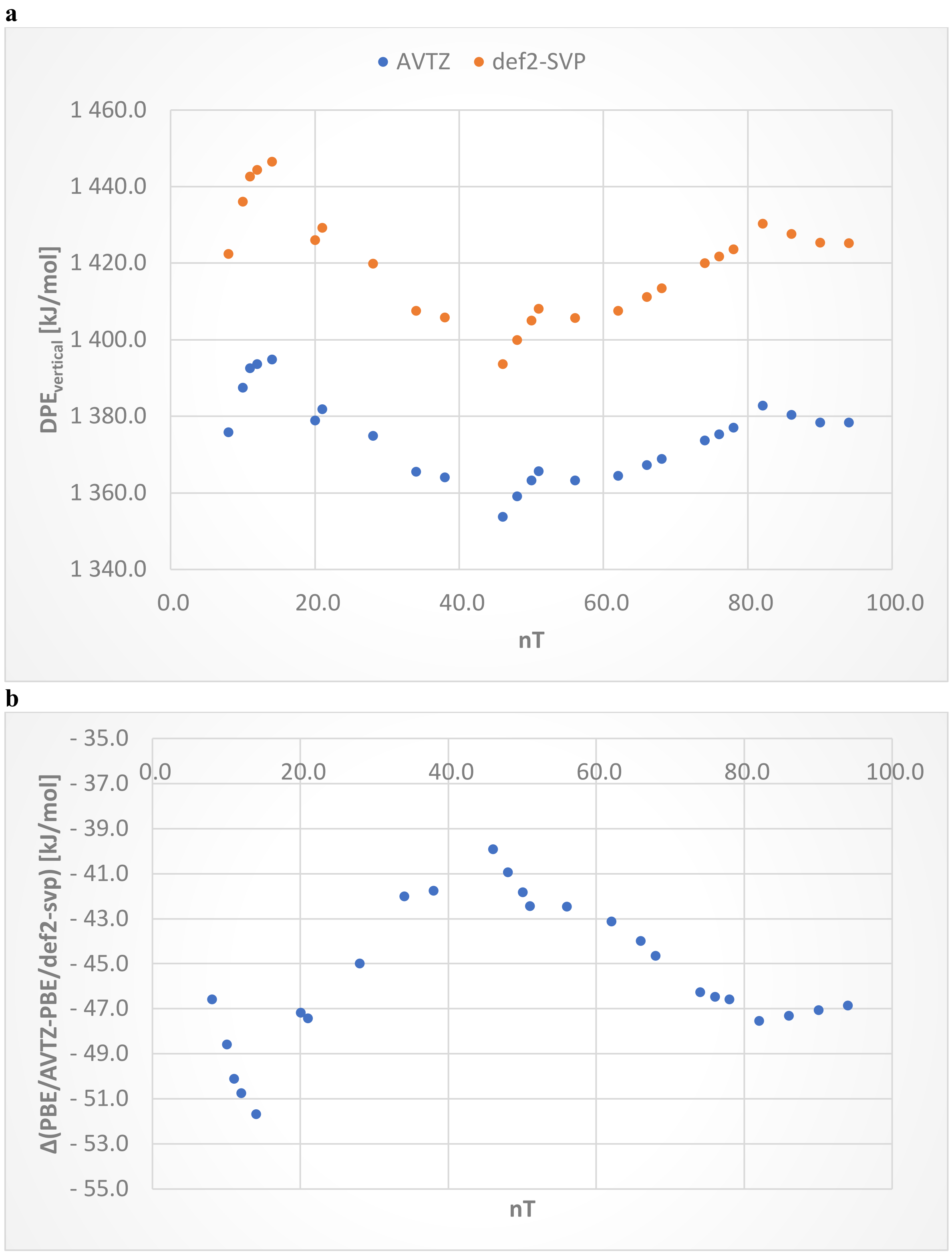

Figure S2 The correlation between DPE as calculated from periodic DFT model (i.e. excluding *ΔBC* from Eq.1) and framework density (FD). For zeolites with more than one T-position the average value of DPE is taken (i.e. random distribution of Al is assumed).

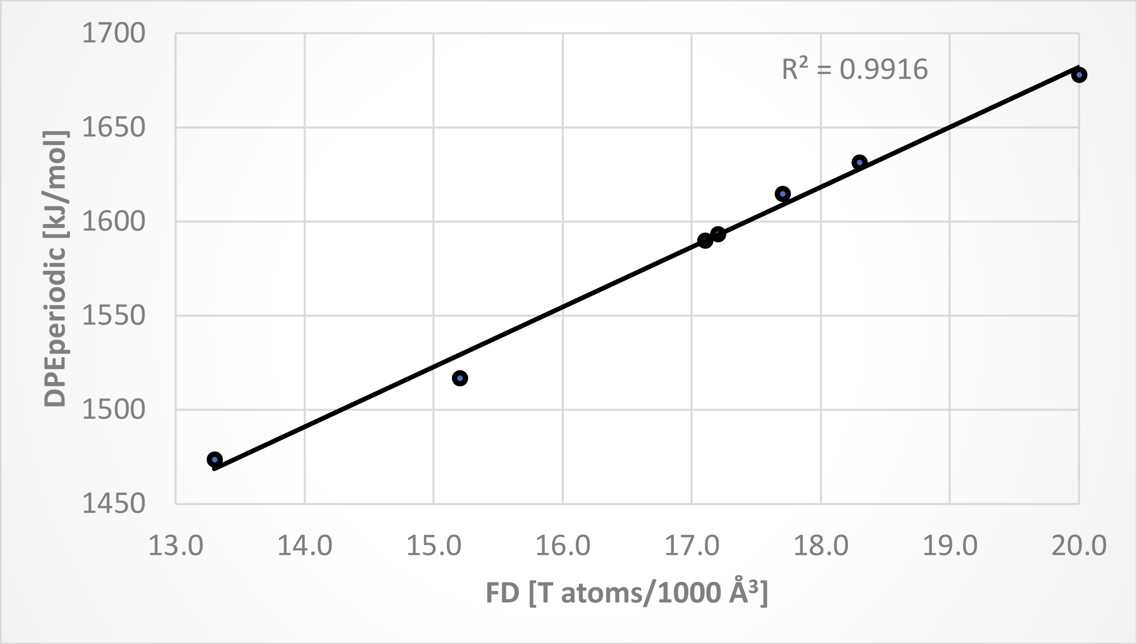

Figure S3 The correlation between periodic deprotonation energies from this work and of that Ref.[1] for most stable BAS sites.

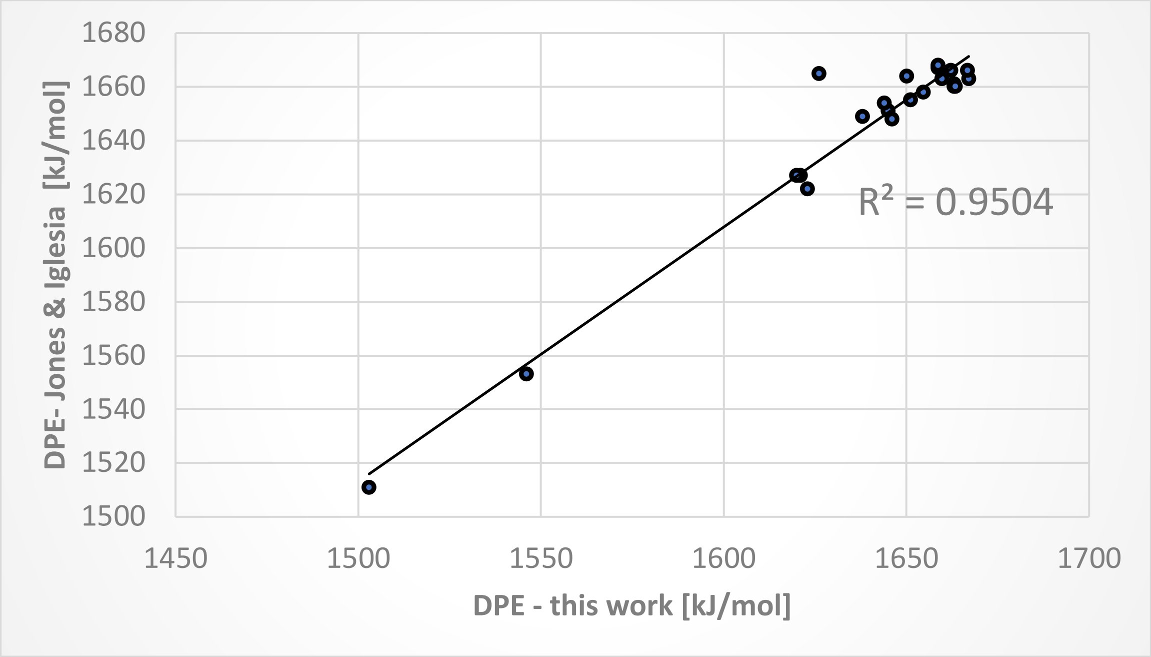

Figure S4 The variance of deprotonation energy dependence of cluster size. For the cluster below 9T, we observed several outlier values (removed) and large fluctuations in variance. Thus, the DPE averaging for clusters is performed from 9T models onward. Also, linear interpolation is used for points that are missing due to the fact that did not fulfill the criteria for cluster construction.

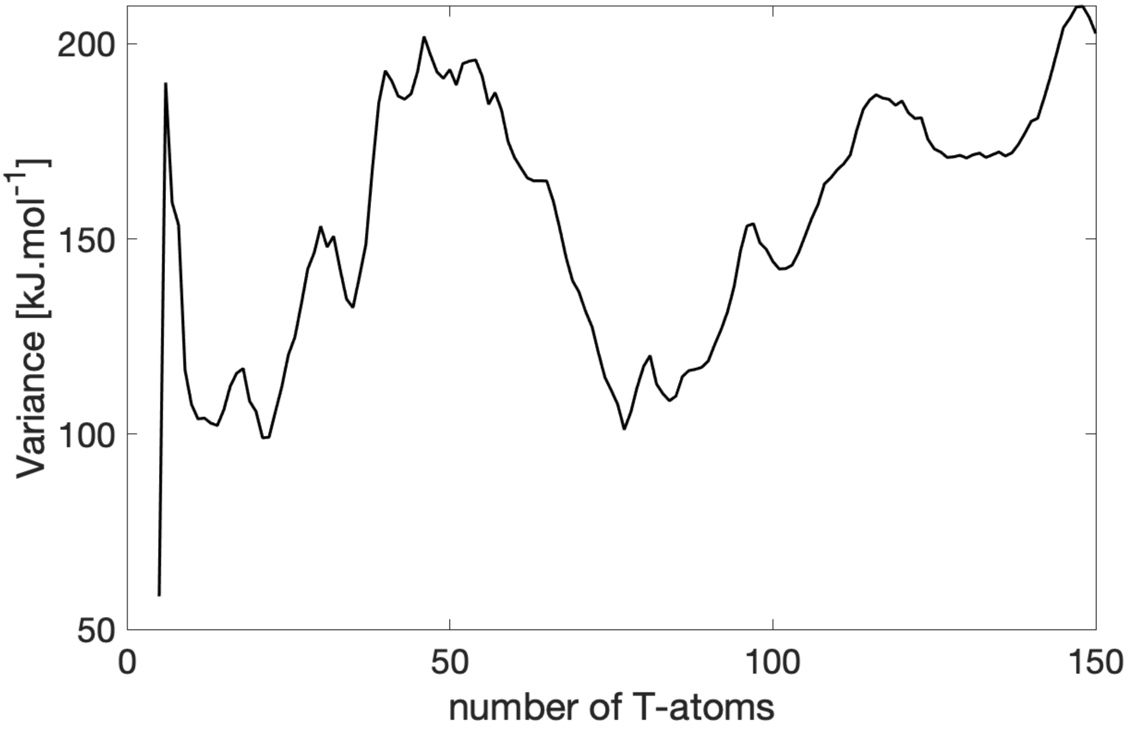

Figure S5 Difference between deprotonation energies of periodic model excluding ΔBC correction and DPE_{model} values (a). The residuals from linear curve are shown in (b). For material with more than one T-position the average values are taken.

a

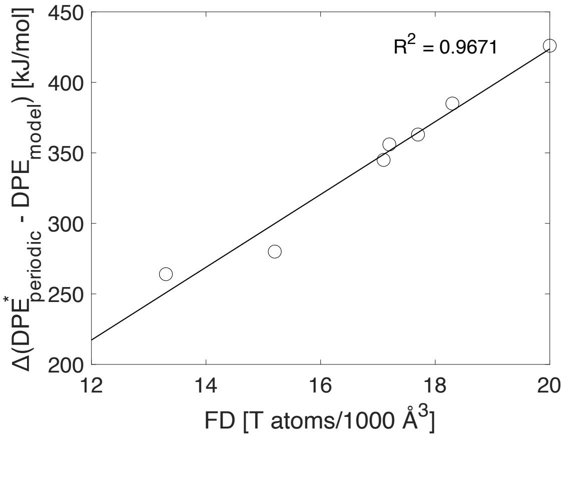

b

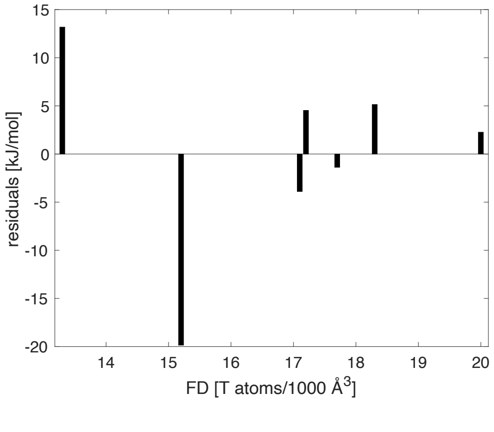

Table S3 Comparison between DPE in this work and some of the QM/MM results in the literature.

Material	T-position	DPE[kJ/mol][a]	
		This work[a]	Literature[2-5]
FAU	T1	1239 (1210)	1169[b],1171[b],1220,1224,1250, 1252
CHA	T1	1266 (1237)	1190[b],1225-1235, 1271,1277
MOR	T4	1268 (1240)	1195[b] ,1274-1281
MFI	T2	1280 (1251)	1279
	T7	1273 (1244)	1200[b], 1205[b], 1230,1281,1286
	T12	1266 (1237)	1275

[a] ΔZPVE corrected data are in parentheses

[b] data corrected for ΔZPVE (-35 kJ/mol) including correction for basis set incompleteness (-46 kJ/mol).

Table S4 Comparison between DPE (in kJ/mol) in this work and QM/MM approach in Ref.[6] The ΔZPVE correction is excluded.

Material	T-position	This work[a]	Ref.[6]
FAU	T1	1239	1210
CHA	T1	1266	1233
MOR	T1	1263	1239
	T2	1265	1245
	T3	1270	1259
	T4	1268	1263
FER	T1	1275	1251
	T2	1284	1249
	T3	1282	1262
	T4	1279	1256
MFI	T1	1273	1244
	T2	1280	1251
	T3	1274	1250
	T4	1283	1250
	T5	1274	1245
	T6	1272	1246
	T7	1273	1242
	T8	1280	1238
	T9	1268	1246
	T10	1267	1244
	T11	1282	1241
	T12	1266	1246

DPE_{model} description

The DPE_{model} for calculation of deprotonation energy employs both cluster and periodic ab initio results (Figure S6). The following scheme was proposed: First, the electrostatic limit of H^+...zeolite$^-$ interaction energy was obtained for every single BAS of Table 1. For that, the electrostatic potential of siliceous zeolite needs to be evaluated at the position of the BAS hydrogen atom (not present) using formal charges and corrected for the Si → Al substitution. This calculated limit is denoted as $-E_{elst}$ and may be obtained by various approaches (e.g. Ewald summation) – we employed the procedure described in Ref. 6. Obviously, this value is overestimated as formal charges were used and need to be scaled down. The scaling factor was fitted on series of clusters to recover the ab initio electrostatic potential from the formal-charges (see Figure S7a and Figure 5). Product of E_{elst} and the scaling factor provides the electrostatic contribution for the "infinite" system.

Second, the "non-electrostatic" contribution of H^+...zeolite$^-$ interaction was evaluated from DFT calculations on cluster models. The difference between ab initio vertical deprotonation energy and ab initio electrostatic potential at the BAS for n-th cluster is defined as follows:

$$\Delta E_n = DPE_n^{vertical} - E_n^{elstat}$$

The ΔE_n yields a fast-converging series that can be easily extrapolated to infinite cluster limit ($\lim_{n\to\infty} \Delta E_n$) via exponential (Figure S7b).

Other contributions to DPE_{model} are the deformation energy and change of the zero-point vibrational energy evaluated on the periodic model using ab initio. The sum of all the mentioned terms (see Figure 3) yields the estimate of deprotonation energy given as DPE_{model} in Table 1 of the main article.

At this point, we would like to mention some technical details of our model. Figure S7a demonstrates how the E_{elst} scaling factor is obtained. The E_{elst} scaling is converged when the root-mean square deviation (rmsd) between the two E_{elst} data sets (ab initio E_{elst} and empirical E_{elst} calculated from formal charges) falls below ~5.7 kJ.mol^{-1} (one unit on pK_a scale). This implies that for each series of model clusters a certain threshold in model cluster size needs to be reached to satisfy the criterium (Table S2). The scaling can be used on average from 59T model cluster onward. It is notable that results are insensitive to the assigned charges for each atomic species, and thus formal charges are sufficient for a given purpose. Furthermore, the

cluster limit energy $\lim_{n\to\infty} \Delta E_n$ is calculated from the cluster size threshold as defined from electrostatic scaling, because the direct electronic effects are largely diminished (Figure S7b)

Figure S6 Schematic representation of the proposed model

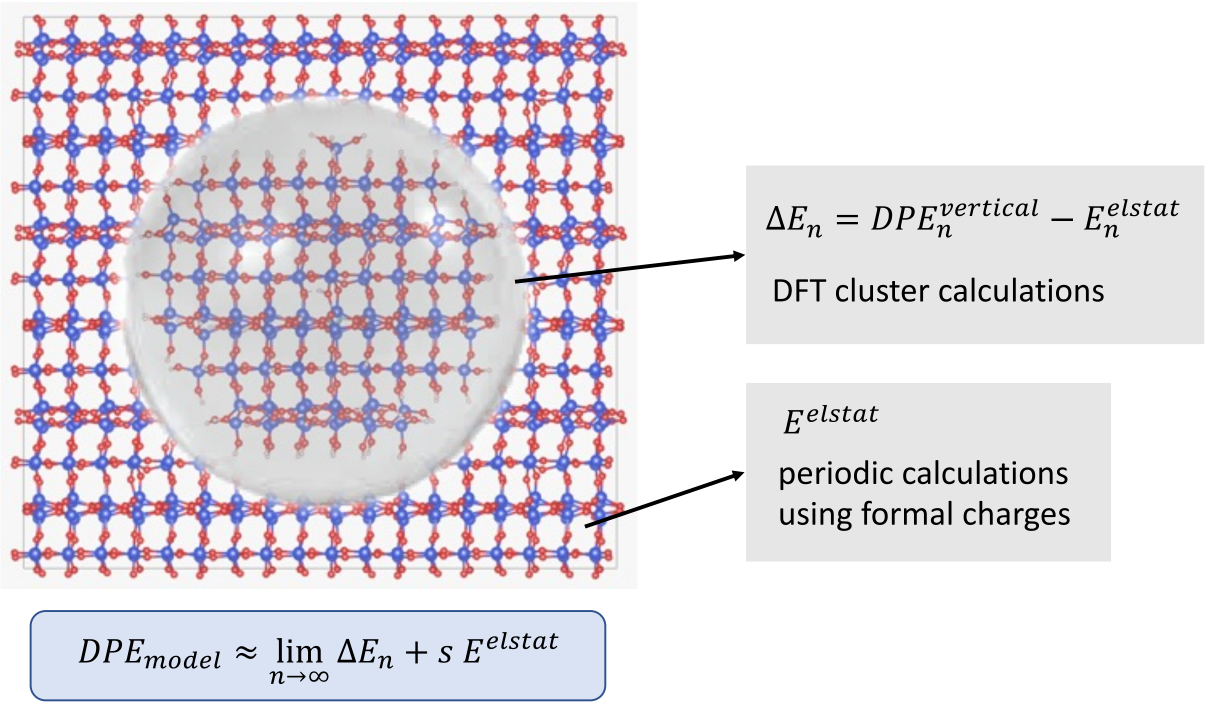

Figure S7 Example of the construction of DPE_{model} for series of **CHA** cluster models (a) difference between ab initio electrostatic potential of anion at the BAS proton and scaled electrostatic potential calculated from formal charges, (b) limiting behavior of cluster energies after subtracting electrostatic contribution of anion at the BAS. The data below cluster size threshold are shown in grey.

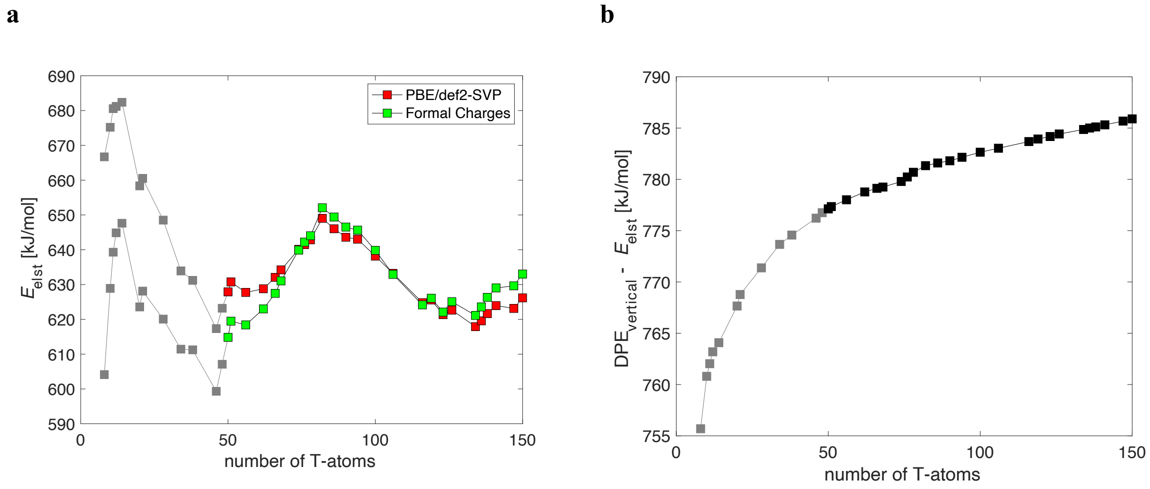

References

1 Jones, A. J. & Iglesia, E. The Strength of Brønsted Acid Sites in Microporous Aluminosilicates. *ACS Catalysis* **5**, 5741-5755, doi:10.1021/acscatal.5b01133 (2015).

2 Sauer, J. & Sierka, M. Combining quantum mechanics and interatomic potential functions in ab initio studies of extended systems. *J. Comput. Chem.* **21**, 1470-1493, doi:10.1002/1096-987X(200012)21:16<1470::AID-JCC5>3.0.CO;2-L (2000).

3 Sauer, J., Schröder, K.-P. & Termath, V. Comparing the Acidities of Microporous Aluminosilicate and Silico-Aluminophosphate Catalysts: A Combined Quantum Mechanics-Interatomic Potential Function Study. *Collect. Czech. Chem. Commun.* **63**, 1394-1408, doi:10.1135/cccc19981394 (1998).

4 Brandle, M. & Sauer, J. Acidity Differences between Inorganic Solids Induced by Their Framework Structure. A Combined Quantum Mechanics/Molecular Mechanics ab Initio Study on Zeolites. *J. Am. Chem. Soc.* **120**, 1556-1570, doi:10.1021/ja9729037 (1998).

5 Eichler, U., Brändle, M. & Sauer, J. Predicting Absolute and Site Specific Acidities for Zeolite Catalysts by a Combined Quantum Mechanics/Interatomic Potential Function Approach. *J. Phys. Chem. B* **101**, 10035-10050, doi:10.1021/jp971779a (1997).

6 Rybicki, M. & Sauer, J. Acid strength of zeolitic Brønsted sites—Dependence on dielectric properties. *Catal. Today* **323**, 86-93, doi:10.1016/j.cattod.2018.04.031 (2019).
